# Complications Following 500 Consecutive Explantations with Simultaneous Breast Lift

**DOI:** 10.1016/j.jpra.2026.04.002

**Published:** 2026-04-09

**Authors:** Stephen C. Nicolaidis, Manu Boucher, Melissa Lu

**Affiliations:** a Division of Plastic Surgery, Department of Surgery, Centre hospitalier de l’Université de Montréal, Université de Montréal, Montreal, Quebec, Canada; b Faculty of Medicine, Université de Sherbrooke, Sherbrooke, Quebec, Canada; c Faculty of Medicine, Université de Montréal, Montreal, Quebec, Canada

**Keywords:** Capsulectomy, Explantation, Breast implant illness, Pneumothorax

Patients with breast implant illness (BII) are requesting complete capsulectomy along with the removal of their breast implants, commonly referred to as explantation. While there are numerous reasons to remove the entire capsule when removing breast implants, there is no question that complete capsulectomy is more complicated than simple removal of breast implants.^1^, ^2^, ^3^ Moreover, Plastic Surgeons who are sceptical of BII argue that complete capsulectomy is too dangerous. Although I have presented on the complications of capsulectomy, these complication rates have never been published.^4^^,^^5^

As a measure of good practice, I noted my complications in my first 500 bilateral capsulectomies performed between January 2019 and February 2022. All patients had either saline or silicone implants, above or below the muscle, bilateral or unilateral, intact or ruptured. Patients with breast reconstruction following cancer resection were excluded. Patient age ranged from twenty-seven to seventy-six years old, while the duration with breast implants ranged from less than a year to forty-six years. Simultaneous breast lifts were performed in 470 of the 500 patients Therefore, I also took note of any nipple compromise.

Patients were explained that removal of the implant with its surrounding capsule as a single specimen could not be guaranteed, but that complete capsulectomy would always be performed using a 11–13 cm incision in the inframammary fold, depending on the size of the implants. Six percent of patients were not felt to require any form of a lift because of good nipple positioning and adequate breast tissue. On the other hand, 94% of patients required simultaneous breast lift, the type of which was determined by the amount of breast tissue, degree of ptosis, nipple size and breast implant size. Forty six percent of all the patients had nipple positioning 1–3 cm too low relative to their inframammary fold and underwent a simultaneous skin-only periareolar lift. Forty eight percent of all patients were felt to require a simultaneous complete breast lift, with the “anchor” scar. Relative indications for complete lift were poor skin quality (thin skin or stretch marks), nipple ptosis greater than 3 cm, very large nipples, implant volumes greater than 400–450 mL and a history of previous complete breast lift. A glandular pedicle separated from the surrounding skin envelope versus a superomedial pedicle were used, depending on the mechanical requirements for raising the nipple. Smokers were told that no lift would be performed unless they completely stopped smoking at least 4 weeks prior to surgery.

Out of the 500 patients who underwent capsulectomies with breast lifts, 25 patients had complications post-operatively (total of 5%). The most common complication was hematoma with a total of nine cases (0.9% per breast).

The lung pleura was punctured 8 times in the first 500 cases. Given that this is the complication of greatest concern-discussion with complete capsulectomies, I focused on this particular complication in my following 500 explantations, during whjch 4 more punctures took place. Therefore, a total of 12 punctures of the lung pleura occurred over 1000 cases (1.2% per breast). There was never a significant pneumothorax nor was hospitalization ever required.

There were three cases of phlebitis (0.6% per case), two cases in the arm which had received intravenous access and one in a leg. One phlebitis in the arm led to a non-life-threatening pulmonary embolus. Three seromas developed (0.3% per breast) which required drainage under ultrasound.

A skin perforation in the upper breast pole occurred in two patients with subglandular implants, representing a risk of 0.2% per breast. The skin perforation resulted from an implant capsule that was directly adherent to the overlying skin. Both cases occurred amongst my first 50 capsulectomies. The first patient had very little breast tissue with a severe capsular contracture, while the second patient was exceptionally muscular.

There was concern for nipple compromise in 3 out of 940 nipples, all of which followed complete lifts. One of the three cases involved a patient on Methotrexate who required an 8 cm raising of the nipple and suffered a significant bleed upon her return home; there was a delay in draining the hematoma and the patient finished with a 25% nipple loss. The other two cases received hyperbaric oxygen treatment and finished with no nipple loss. Therefore, there was a 0.1% risk of nipple necrosis per breast.

Given our lack of understanding regarding BII as well as the full significance of the capsule forming around breast implants, it is important for Plastic Surgeons to fully inform patients of all the risks and benefits of capsulectomy in order for them to make an informed decision themselves. The complication rates here can help to clarify those risks.

## Declaration of competing interest

None declared.
